# Spatialization of Time in the Entorhinal-Hippocampal System

**DOI:** 10.3389/fnbeh.2021.807197

**Published:** 2022-01-06

**Authors:** Troy M. Houser

**Affiliations:** Department of Psychology, University of Oregon, Eugene, OR, United States

**Keywords:** place cells and time cells, grid cells, concept cells, temporal context, cognitive maps, hippocampus, entorhinal cortex

## Abstract

The functional role of the entorhinal-hippocampal system has been a long withstanding mystery. One key theory that has become most popular is that the entorhinal-hippocampal system represents space to facilitate navigation in one’s surroundings. In this Perspective article, I introduce a novel idea that undermines the inherent uniqueness of spatial information in favor of time driving entorhinal-hippocampal activity. Specifically, by spatializing events that occur in succession (i.e., across time), the entorhinal-hippocampal system is critical for all types of cognitive representations. I back up this argument with empirical evidence that hints at a role for the entorhinal-hippocampal system in non-spatial representation, and computational models of the logarithmic compression of time in the brain.

## Introduction

It has been almost 20 years since the first report of “concept cells,” following single unit recordings of cells in the medial temporal lobe that fired selectively to different concepts (Quiroga et al., 2005). Specifically, these cells fired in response to participants being presented with pictures referring to the same concept, even when the pictures shared no sensory information (Quiroga et al., 2005; Quiroga, 2012; Rey et al., 2020; Quian Quiroga, 2021). From these data, it has become clear that structures in the medial temporal lobe, such as the hippocampus and entorhinal cortex, do not only care about representing the environment. Traditionally, this is the function that the hippocampus and surrounding cortices have been proposed to have. In this Perspective, I will first explain how neuroscience and psychology came to that conclusion. Then, I will briefly review nascent findings for non-spatial representation in the entorhinal-hippocampal system. Finally, I will introduce the idea that cognitive maps depend on temporal continuity by bridging the philosophy of Henri Bergson and computational models of logarithmic time in the brain.

## What is a Cognitive Map?

The idea that the hippocampus and surrounding cortices represent the environment emerged from the seminal work of Edward Tolman, who showed that rats could make accurate spatial inferences. For example, in the sunburst maze experiment of Tolman et al. (1946a,b), rats were given access to a single path that changed direction three times before leading to a goal box. Importantly, the goal box was close to the starting point but could only be reached *via* the roundabout path. Then, the authors blocked the former path and introduced 18 new paths, one of which led directly to the goal box. Most rats chose correctly, despite having no experience with these new paths. Tolman (1948) suggested that animals form representations of the spatial layout—cognitive maps—to flexibly navigate their surroundings.

Neurophysiological support for cognitive maps came with the discovery of place cells in the hippocampus (O’Keefe and Dostrovsky, 1971) that fire when an animal occupies a specific spatial location. Serial activation of place cells was found to represent adjacent places (Muller and Kubie, 1989), making them a likely candidate for facilitating navigation (Buzsáki and Moser, 2013). Thus, O’keefe and Nadel (1979) posited that the hippocampus is the neurophysiological instantiation of cognitive maps. This sparked considerable debate as to what a cognitive map actually is. At the time, many treated a cognitive map as equal to other stimuli (Estes, 1955a,b, 1960; Rescorla, 1967, 1968, 1969), but this view quickly fell out of favor in light of findings that behavior could become conditioned to background cues (Jones and Skinner, 1939; Penick and Solomon, 1991; Phillips and LeDoux, 1992, 1994; Pavlov, 2010), or tonic stimuli (Konorski, 1967; Flynn, 1969). However, this still begged the question, “Which background cues?”

The radial arm maze experiment (Olton and Samuelson, 1976; Olton, 1987) provided evidence that it was not specific stimuli that induced cognitive map-like representation in the brain, but the spatial relationships between stimuli, or the global configuration (Chun and Jiang, 1998). The radial arm maze consists of a circular chamber with eight arms extending from it. At the end of each arm is a food reward. Attached to the walls just beyond the ends of the arms are (extramaze) visual cues. Rats are tested for efficiency in completing the maze, which consists of collecting each reward. Thus, the most efficient route is to visit each arm once and none repeatedly. As a result, this task relies on memory for which arms were previously visited. When the visual cues are rotated, so that each cue maintains its relationship with all the other cues, performance in the maze does not change; however, when the cues are transposed (i.e., when the spatial relations change), performance is disrupted (Suzuki et al., 1980). These studies revealed both that cognitive maps are critical for memory and that they are constituted by relationships between stimuli.

If the hippocampus, which supports cognitive map learning, creates global configurations of any set of stimuli, why would cognitive maps be made only for spatial environments? That is, the brain makes connections *via* Hebbian learning (Attneave and Hebb, 1950), which is associative. If associations in the brain can represent spatial relations, they should also be able to represent other types of relations. Indeed, evidence implicates the hippocampus in encoding these associations. One study found greater hippocampal activity for remembering correct details associated with words (Eldridge et al., 2000). Another study found that hippocampal activation increased when participants associated a person with a house (Henke et al., 1997). Face-name associations have been shown to elicit greater hippocampal subfield activation (Zeineh et al., 2003). These are just a few examples of the hippocampus associating non-spatial information. Perhaps, the hippocampus cares more about associations in general than spatial relations in particular (Eichenbaum et al., 1992, 1994, 1999, 2007; Eichenbaum, 2004). This notion is consistent with the hippocampal index theory (Teyler and DiScenna, 1986; Teyler and Rudy, 2007; Goode et al., 2020) that says that the hippocampus is a content-free index of associations between sensory information supported by the neocortex. Thus, the hippocampus can support spatial, temporal, conceptual, etc., relations (Eichenbaum, 2014, 2017a,b,c; Howard et al., 2014; Howard and Eichenbaum, 2015; Buzsáki and Tingley, 2018). Empirical data supporting this notion shows that transitive inferences depend on the hippocampus (Dusek and Eichenbaum, 1997). Transitive inference is a form of deductive reasoning that allows one to infer A < C from learning A < B and B < C. It is also a form of higher-order conditioning (Bouchekioua et al., 2021), initially shown by Pavlov (2010) and Brogden (1939). To illustrate, a dog may learn that a bell signals food. Then, the food is replaced with a light. Finally, the light alone is shown and the dog salivates, despite having never experienced the light with the food. Remarkably, transitive inference can explain cognitive maps without any recourse to spatial relations. For example, in the sunburst maze experiment, rats traversed three arms before reaching the goal box (Tolman et al., 1946a,b). Each of these arms can be represented as a vector encoded by a population pattern of neural firing rates (Pouget et al., 2003). Thus, computing the location of the goal box relative to the starting point becomes a matter of simple vector addition and subtraction, which is also the basis for path integration (Mittelstaedt and Mittelstaedt, 1980; Wehner and Srinivasan, 1981; Müller and Wehner, 1994; Blair et al., 1997; Sharp, 1999; Etienne and Jeffery, 2004; McNaughton et al., 2006; Sharp and Koester, 2008; Savelli and Knierim, 2019; Bicanski and Burgess, 2020; Stangl et al., 2020; Bouchekioua et al., 2021).

If aspects of the world (i.e., single variables) can be represented by the activity of a population of neurons (Pouget et al., 2000, 2003; Panzeri et al., 2015; Saxena and Cunningham, 2019; Ebitz and Hayden, 2021), there is no reason to assume that those aspects have to be spatial. Indeed, a significant number of recent studies have found that the entorhinal-hippocampal system is critical for representing conceptual information, as anticipated by Quiroga et al. (2005). Specifically, the entorhinal-hippocampal system enacts a transformation of categorical to dimensional data, making this system the locus for prototypical knowledge (Zeithamova et al., 2012; Bowman and Zeithamova, 2018; Mack et al., 2018; Bowman et al., 2020). This category-to-dimension transformation idea is akin to the hippocampus as an associator of discontiguous events (Wallenstein et al., 1998), which has been largely confirmed with the discovery of time cells (MacDonald et al., 2011; Kraus et al., 2013; Eichenbaum, 2014; Salz et al., 2016). Time cells fire during the temporal gaps between salient events. Specifically, their activity tiles the gap to create evenly spaced timestamps.

Evaluating information within the context of a representational dimension is what enables us to make inferences, generalize, and abstract. It is at the heart of higher-order cognition. Dimensions require metrics so that information can be assessed relative to dimension axes. Since their discovery, grid cells have been posited to form a metric space in the medial entorhinal cortex (McNaughton et al., 2006; Giocomo et al., 2011; Dang et al., 2021). Grid cells fire at the vertices of tessellating, equilateral triangles representing space (Hafting et al., 2005). Like the hippocampus, however, grid cells are also not specifically tuned for space, as their properties have been described with computational principles (Dordek et al., 2016; Stachenfeld et al., 2017; Baram et al., 2018; Behrens et al., 2018; Gershman, 2018; Momennejad and Howard, 2018; Bicanski and Burgess, 2019; Mark et al., 2020; Momennejad, 2020; Rueckemann et al., 2021), specifically, as a (basis) set of vectors that can linearly combine to represent any point in two dimensions. This “grid code” has since been found to facilitate non-spatial representation, such as for virtual (Doeller et al., 2010), imagined (Bellmund et al., 2016; Horner et al., 2016), conceptual (Constantinescu et al., 2016), visual (Julian et al., 2018; Nau et al., 2018), odor (Bao et al., 2019), egocentric (Moon et al., 2020), social (Park et al., 2021), semantic (Viganò et al., 2021), and contextual (Julian and Doeller, 2021) information in humans.

Consider the recent study by Park et al. (2021), which also revealed that the grid code helps to make inferences. Participants learned pairs of 16 faces (AB) and how they compared along the dimension of popularity (e.g., A < B). Then, they learned how the same faces differed along the dimension of competence. Together, the faces formed a 4 × 4 grid that differed along popularity and competence axes (i.e., dimensions). While scanned, participants were shown a face and subsequently shown a pair of faces with the task of inferring which of the pair would make a better business partner for the first face. When comparisons featured an angle between faces on the 4 × 4 grid that aligned with the grid axes of grid cell activity, the entorhinal cortex displayed significant modulation. These findings indicate that participants used the grid code to navigate the non-spatial (i.e., social) cognitive map.

## What is Temporal Context?

The entorhinal-hippocampal system thus seems to only worry about space because it is worried about continuity, and our construct for space is intimately bound up with continuity. However, it is important to note that space, as it is experienced, is discontiguous. Everywhere we turn, we will see the sharp contours outlining objects. To imagine an empty and homogenous container in the absence of all objects is exactly that: imagination. Instead, continuity can be derived from time, which is an experienced phenomenon. Moreover, time can be accurately inferred by measuring the accumulation of changes along representational dimensions. I suggest that the brain co-opts its computational mechanisms used to represent the passage of time to represent dimensions of any type of information. Firstly, how does the brain represent time?

**FIGURE 1:**
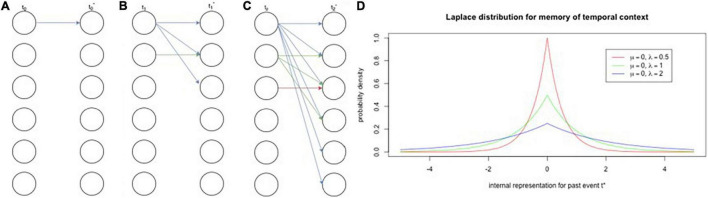
Encoding and retrieval of events in neural population activity *via* temporal context cells. **(A–C)** Encoding. Panels **(A)** through **(C)** depict the exponential diffusion of stimulus traces through a population of neurons. Within each panel, the left column is the stimuli active at a given timestep (t0–t2) and the right column is an internal representation of that time step (t0*–3*). Each node indicates a stimulus and arrows connect stimuli to their internal representations as represented by neural populations. If we focus on just the blue trace [i.e., the first stimulus, present in panel **(A)**], we see that is spreads out, or diffuses with time, contacting more and more nodes with each time step. The same diffusion process occurs for the green trace, but to a lesser extent given that it was only presented at the second time step [panel **(B)**]. Critically, with the diffusion of a stimulus trace comes weaker individual traces, which corresponds to equal areas under their distributions. **(D)** Retrieval. Panel **(D)** depicts probability densities for three populations of neurons each with varying exponential decay rates. Thus, the blue trace from panels **(A–C)** is more spread out and causes activation at time 0 (the present moment) with low probability. Inset shows location and scale parameters (mu and lambda, respectively). A crucial prediction that this distribution makes is that, as time shifts away from 0, the traces with longer decay rates will have higher probabilities for activation, which has been shown in hippocampal time cells that have wider time fields later in a delay (Salz et al., 2016).

Shankar and Howard (2010, 2012) put forth a model that consists of two feedforward neural network layers that may explain how the brain represents dimensional information, such as intensity, time, space, and, as discussed, concepts. The authors label each layer, respectively, as the timing mechanism and the associative learning mechanism. The timing mechanism is initiated by a vector of neural activity that activates a sequence of leaky integrators (Jaeger et al., 2007), or population of cells that have varying time constants (i.e., rates of decay; Figure 1). These cell populations are similar to filters for exponentially decaying frequencies, whose activity enacts the Laplace transform (Shankar and Howard, 2010, 2012; Howard et al., 2014, 2015a,b; Howard and Shankar, 2018). Recent studies have confirmed the existence of the timing mechanism with what have been deemed “temporal context cells” in the entorhinal cortex (Tsao et al., 2018; Bright et al., 2020). The associative learning mechanism performs the inverse Laplace transform to create, essentially, a number line of time, represented by time cells whose receptive fields are logarithmically compressed (Howard, 2018; Howard and Shankar, 2018). Logarithmically compressed representations of time have been found in hippocampal time cells (Cao et al., 2021).

The logarithmic compression of time is intuitive for at least two reasons: (1) it is reflective of the Weber-Fechner law that is ubiquitous in perception, and (2) it is an efficient way to represent dimensionality. The Weber-Fechner law says that there is a logarithmic relationship between stimulus intensity and perception (J and Fechner, 1889). For example, it is easier to discriminate 1 dot from 2 dots than 100 dots from 101 dots, despite their differences being the same. For episodic memory, this means that there will be greater resolution for things that happened in the recent past than the distant past. Moreover, logarithms themselves are an efficient way to represent large quantities, such as dimensions, which are infinitely large. Breaking a number down by its base conveys this idea clearly: 967 = (9 × 10^2^) + (6 × 10^1^) + (7 × 10^0^). Here, 967 is represented using base ten means, where base corresponds to the value of each digit. Thus, each temporal context cell has a base that corresponds to the width of its receptive field (for time), i.e., its time constant or decay rate. According to Shankar and Howard (2010, 2012), there should be more temporal context cells with narrow receptive fields centered on the present, corresponding to greater representational resolution, analogous to the clustering of cells with narrow tuning curves in the fovea (Howard, 2018; Howard and Hasselmo, 2020). Notably, the population of temporal context cells supporting a representation is *a priori* indicative of an event’s timing. The same way that retinal activity *a priori* indexes location in space by way of the organization of retinal ganglia, the decay rates of temporal context cell activity indexes time. This means that *what* and *when* information is decodable from the entorhinal cortex.

While this theory of temporal context is still in its infancy, a number of predictions can be made according to its computational principles. For example, in this framework, time becomes congruent with distance, and as such, spatial representations should have more cells underpinning nearby locations. Indeed, border cells, found in the medial entorhinal cortex fire maximally when animals are close to the boundary of an environment, with rates that decay with increasing distance from said boundary (Solstad et al., 2008). The current theory predicts that these firing rates decay exponentially with distance. This distribution of time coverage by individual temporal context cells has also been observed in place cells (Mizuseki et al., 2012). How temporal context fits with the work surrounding theta cycles is an interesting next step as well. That is, what are the differing predictions made by either mechanism for time? Can one mechanism subsume the other in explanatory power? An interesting hypothesis by Foster and Wilson (2006) suggests that reverse replay, which is associated with theta sequences, is associated with a gradually decaying dopamine signal. This idea could potentially link temporal context with decision-making and reward seeking behavior. Moreover, head direction cells which are crucial for the brain’s navigation system may control attention to a specific location along the internal timeline that the hippocampus constructs. This hypothesis is highly speculative but nevertheless reflects the head direction cell function of orienting in (abstract) space (Bicanski and Burgess, 2020). Finally, a recent study found that unipolar brush cells in the cerebellum adhere to logarithmically compressed basis functions associated with a gradient of metabotropic signaling (Guo et al., 2021), suggesting that regions outside the medial temporal lobe that must keep track of time may utilize similar mechanisms (Ranck, 1979). This diverse range of applications of the logarithmic compression of time suggests that all dimensional data—spatial, temporal, conceptual, etc.,—is governed by the same computational principles.

## The Spatialization of Time in the Brain

**FIGURE 2:**
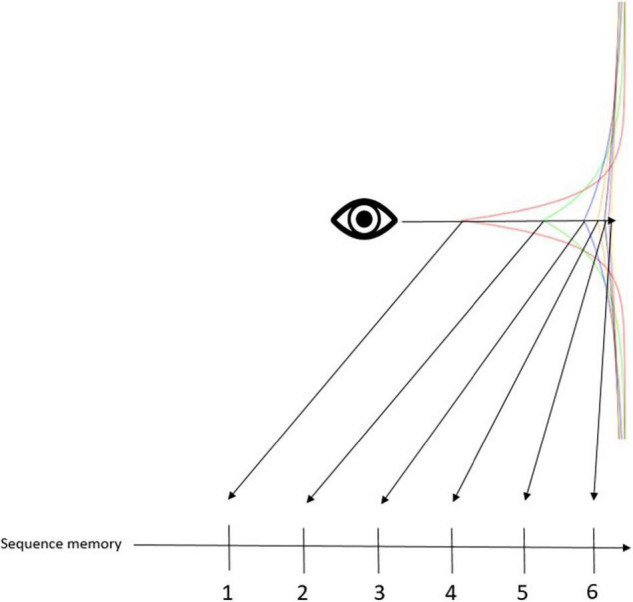
Conceptual framework for the associative mechanism. This figure is meant to illustrate the associative mechanism of temporal context. Specifically, we have a Laplace distribution of six different exponentially decaying stimulus traces. The eye icon represents the mind’s eye that looks back on an event that happened in the past. The arrow cutting across the different stimulus traces shows how the Weber-Fechner law manifests in a Laplace distribution: stimulus traces further in the past become clustered closer together, making it more difficult to tell them apart. This distribution is converted to a rank order of sequential memory, enacted by time cells. Rank order can be computed a number of different ways, one of which would be to compute the similarity between each trace and the present (or, meaningful zero marker). Thus, in the figure, traces further from the eye indicate greater dissimilarity, and are thus ranker further in the past.

Assuming that the entorhinal-hippocampal system converts the logarithmic relationship among temporal context cell activity into the serial positions of time cells, it makes sense that the same system supports cognitive map learning. Logarithmically compressed time in the brain enables representation of Bergson; Bergson; Bergson’s (1889; 1908; 1911) *duration*. *Duration* was a construct that the philosopher, Henri Bergson, formulated to distinguish phenomenological time from mathematical time. Mathematical time is a succession of points. There is always a gap between the points, no matter how small, which Bergson argued cannot convey the continuity of bodily experience. As we have seen, our brains manage to simulate continuity *via* transforming temporal context into serial order. This notion can also be represented with a hierarchical (Bayesian) framework, where higher levels contextualize lower levels (Kiebel et al., 2008; Ramstead et al., 2018; Badcock et al., 2019; Friston, 2019; Cao et al., 2021). In this framework, like the timing mechanism of Shankar and Howard (2010, 2012), time is not only successive because each succession is reflective of (i.e., contextualized by) the temporal context that gradually drifts. The computational principles of duration can be used for any continuous representation, e.g., cognitive maps. Call this the “spatialization of time” (Bergson, 1889; Buonomano and Maass, 2009; Buonomano, 2017). The spatialization of time corresponds to treating any timepoint as if it were in the present, so that all timepoints can be represented simultaneously. In other words, it is the idea that things that occurred in succession can be represented in the brain simultaneously. The present becomes a “meaningful zero,” by which the brain can measure representational distance (Figure 2). More dissimilarity means greater distances. Then, dissimilar representations get ranked by associating with time cells that enact serial position. This associative mechanism is a simple chaining tool that reflects discrete successions that nevertheless maintains the continuous (i.e., logarithmically compressed) structure in the background. Discrete successions are of course more easily recognized, and hence, serve many adaptive functions, which is probably why our brain adopted such computational strategies. Moreover, this systematic transformation of phenomenological time to mathematical time in the brain can be used for any continuous information. That is to say that cognitive maps (Tolman, 1948; O’keefe and Nadel, 1979; Behrens et al., 2018), cognitive spaces (Gärdenfors, 2015; Bellmund et al., 2018), transitive inference (Wallenstein et al., 1998; Eichenbaum, 2014), memory indexes (Teyler and DiScenna, 1986), concept cells (Quiroga et al., 2005; Quiroga, 2012), and cognitive representations in general are all underpinned by the temporal context becoming spatialized in the entorhinal-hippocampal system.

## Concluding Remarks

Since the discovery of concept cells by Quiroga et al. (2005), the capacity of the entorhinal-hippocampal system for cognitive representation has been a perplexing issue. The search for a unifying theory to explain this system’s function has an even richer history. The discovery of place cells led to its hypothesized role in spatial representation, which remains the dominant view. However, relational learning, such as transitive inference, has become a solid contender. Further, concept, or prototype, learning may become popular soon, given the growing evidence of this system’s involvement in navigating so-called conceptual spaces. Subsuming, but not necessarily refuting, all of these hypothesized roles is that of transforming continuous time into successive time points. This idea was first put forth by Henri Bergson, who was a forerunner to the phenomenological philosophies of Husserl, Heidegger, and Merleau-Ponty. Since then, behavioral and computational neuroscience has provided substantial support for such a transformation occurring in the entorhinal-hippocampal system. This framework has the advantage of unifying decades of medial temporal lobe research with psychophysical theories of perception.

## Data Availability Statement

The original contributions presented in the study are included in the article/supplementary material, further inquiries can be directed to the corresponding author/s.

## Author Contributions

TH conceived and wrote the manuscript.

## Conflict of Interest

The author declares that the research was conducted in the absence of any commercial or financial relationships that could be construed as a potential conflict of interest.

## Publisher’s Note

All claims expressed in this article are solely those of the authors and do not necessarily represent those of their affiliated organizations, or those of the publisher, the editors and the reviewers. Any product that may be evaluated in this article, or claim that may be made by its manufacturer, is not guaranteed or endorsed by the publisher.
